# Inhibition of protein phosphatase-1 and -2A by ellagitannins: structure-inhibitory potency relationships and influences on cellular systems

**DOI:** 10.1080/14756366.2018.1557653

**Published:** 2019-01-30

**Authors:** Zoltán Kónya, Bálint Bécsi, Andrea Kiss, Dániel Horváth, Mária Raics, Katalin E. Kövér, Beáta Lontay, Ferenc Erdődi

**Affiliations:** a Department of Medical Chemistry, Faculty of Medicine, University of Debrecen, Debrecen, Hungary;; b MTA-DE Cell Biology and Signalling Research Group, Faculty of Medicine, University of Debrecen, Debrecen, Hungary;; c Institute of Chemistry, University of Debrecen, Debrecen, Hungary

**Keywords:** Tellimagrandin I, mahtabin A, protein phosphatase-1, protein phosphatase-2A, synaptosomal exocytosis

## Abstract

Several ellagitannins inhibited the activity of protein phosphatase-1 (PP1) and -2 A (PP2A) catalytic subunits (PP1c and PP2Ac) with preferential suppression of PP1c over PP2Ac. The inhibitory potency for PP1c followed the order of tellimagrandin I > mahtabin A > praecoxin B > 1.2-Di-O-galloyl-4.6-(S)-HHDP-β-D-glucopyranose > pedunculagin with IC_50_ values ranging from 0.20 µM to 2.47 µM. The interaction of PP1c and tellimagrandin I was assessed by NMR saturation transfer difference, surface plasmon resonance, isothermal titration calorimetry, and microscale thermophoresis based binding techniques. Tellimagrandin I suppressed viability and phosphatase activity of HeLa cells, while mahtabin A was without effect. Conversely, mahtabin A increased the phosphorylation level of SNAP-25^Thr138^ and suppressed exocytosis of cortical synaptosomes, whereas tellimagrandin I was without influence. Our results establish ellagitannins as partially selective inhibitors of PP1 and indicate that these polyphenols may act distinctly in cellular systems depending on their membrane permeability and/or their actions on cell membranes.

## Introduction

It is now well accepted that the phosphorylation of proteins is an important regulatory device in many cellular processes and it is regulated by not only the phosphorylating protein kinases but the dephosphorylating protein phosphatases as well^1^. Protein phosphatase-1 (PP1) and -2 A (PP2A) are two major representatives of the phosphoserine/threonine (P-Ser/Thr) specific enzymes and they are believed to be responsible for the dephosphorylation of more than 90% of P-Ser/Thr side chains in cellular phosphoproteins^2^. PP1 and PP2A occur in cells in many holoenzyme forms in which the catalytic subunits (PP1c and PP2Ac) are associated with distinct regulatory proteins. The three major isoforms (α, β/δ, and γ) of PP1c (*PPP1CA, PPP1CB, and PPP1CG*) may be complexed to close to 100 regulatory proteins which generally include a PP1c-interacting sequence termed the RVxF motif^3^. In the PP2A holoenzymes the core unit consists of PP2Ac (*PPP2CA or PPP2CB*) and a 65 kDa A subunit (termed PP2A-AC) and this dimer is associated with distinct classes of B subunit forming various trimer holoenzymes (PP2A-ABC)^4^.

Serious efforts have been made to distinguish between PP1 and PP2A in biochemical and even more importantly in cellular assays in order to establish their physiological roles by targeting the catalytic centre and the substrate binding regions of PP1c or PP2Ac with inhibitory compounds. In this regard, a number of naturally occurring toxins (okadaic acid, calyculin A, tautomycin, microcystin, etc) have been identified as potent inhibitors of PP1 and PP2A^5^. Although, partial selectivity of several toxins (such as okadaic acid and tautomycin) in the inhibition of PP1 and PP2A are proven *in vitro*, there are still concerns with respect to their specificity in cellular systems^6^ due to their differences in membrane permeability and intracellular concentrations. Nevertheless, calyculin A and tautomycin have been shown to specifically inhibit PP2A and PP1 in Balb/c 3T3 cells^7^, respectively, and this distinction between PP1 and PP2A by these toxins was also confirmed in THP1 leukemic^8^ and endothelial^9^ cells by phosphatase activity assays. Even though, the use of toxins to differentiate between the cellular actions of phosphatase types is limited due to cytotoxicity of these compounds. Thus, search for phosphatase inhibitory molecules has not ceased and still tremendous attempts are made to identify novel ones or increase the selectivity of known inhibitors. With the latter regard, a recent publication^10^ has shown that conversion of microcystin by chemical methods leads to a series of analogues, which have higher selectivity toward PP2A than PP1.

It was reported earlier that gallotannins inhibited both PP1 and PP2A with higher preference of suppression of PP1 over PP2A *in vitro* and cellular effects were also reflected in increased phosphorylation of intracellular proteins^11^. In another context we have shown that other polyphenolic molecules, such as the gallotannic penta-O-galloyl-β-D-glucose (PGG) or epigallocatechin-3-gallate (EGCG) and its derivatives inhibit both PP1c and PP2Ac, but they are more potent inhibitors of PP1c than that of PP2Ac^12^. The analysis of the interaction of PP1c with PGG or EGCG by NMR saturation transfer difference suggested that these molecules, at least in part, exert inhibitory potency on PP1c via interacting with the hydrophobic substrate-binding groove. Both PGG and EGCG suppressed viability of HeLa cells, but these effects might be related to their phosphatase inhibitory feature in part only as polyphenolic compounds have multiple cellular targets, therefore other mechanisms could not be excluded either. There is also an apparent controversy concerning the cellular effects of EGCG: it inhibits phosphatase activity of various cell lines at quite high (100–500 µM) concentration^13^ while it activates PP2A at 5–20 µM concentrations in a laminin-receptor mediated manner^14^. As a consequence, PP1-type myosin phosphatase is also stimulated by PP2A driven dephosphorylation of its myosin phosphatase target subunit-1 (MYPT1) at phosphorylation sites inhibitory on PP1c^9^ suggesting a specific interplay of PP1 and PP2A in cellular phosphatase activation.

**Figure 1.**
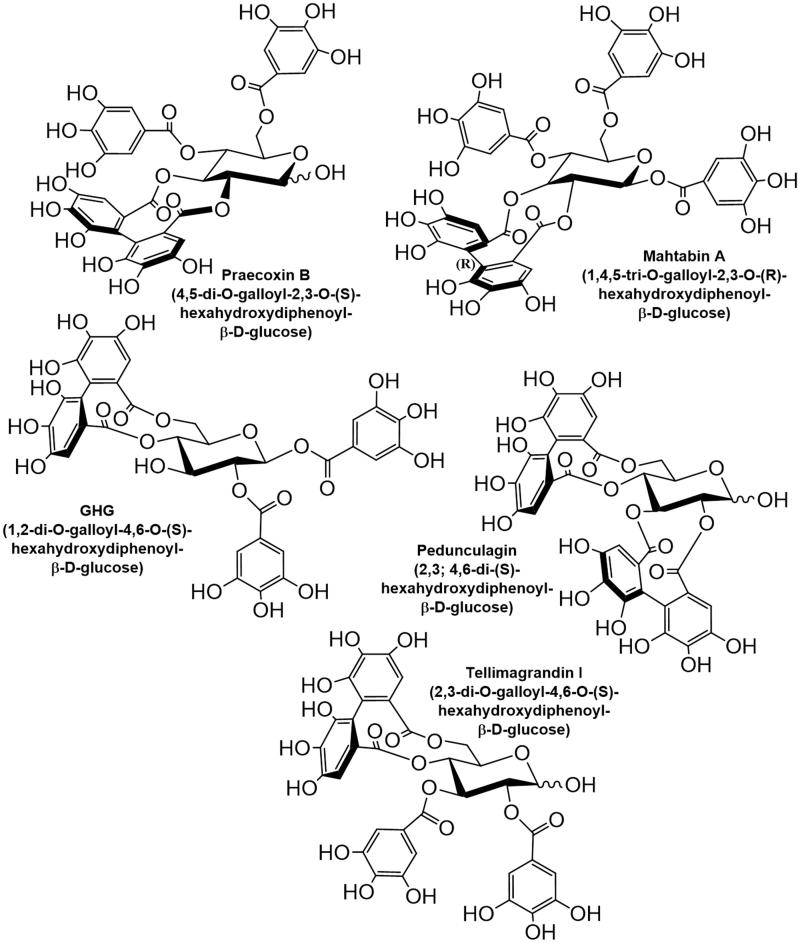
Structure and names of the ellagitannins tested in this study.

The above data indicate that the influence of polyphenolic compounds on protein phosphatases *in vitro* and *in vivo* are quite complex which deserves further attentions in at least two respects that are aimed in our present study: (i) to assay the relationship between the structure of the ellagitannin family of polyphenols^15^ and their phosphatase inhibitory potency. Ellagitannins have been considered major components of plants and fruits with antioxidants, antiviral, and anticancer activities^16^; therefore we aim: (ii) to find the relevance of the physiological influence of polyphenols to their phosphatase inhibitory potency. We assayed five ellagitannins (structure and names are shown in Figure 1) on the activity of PP1c and PP2Ac, and the cellular effects of some of these derivatives were also analysed. Our results suggest that there is a structural dependence of the phosphatase inhibitory features of ellagitannins, and their selectivity for inhibition of PP1 over PP2A is quite high. Moreover, we also found that the two most potent inhibitors (tellimagrandin I and mahtabin A) have diverse effects on the survival of HeLa cells and on the exocytosis of cortical synaptosomes.

## Materials and methods

### Chemicals

Tellimagrandin I was purchased from Nacalai Tesque Inc. (Kyoto, Japan). An initial sample of tellimagrandin I^17^, praecoxin B and mahtabin A^18^, pedunculagin^19^, and 1,2-di-O-galloyl-4,6-(S)-hexahydroxydiphenoyl-β-d-glucose (GHG)^20^ were synthesised, isolated and characterised as described in the respective references and kindly provided by Dr. Karamali Khanbabaee and Dr. Tibor Kurtán (Department of Chemistry, University of Debrecen, Hungary).

### Materials

Materials were obtained from the indicated sources. Anti-SNAP-25^pThr138^ (Abgent, San Diego, CA, USA), HeLa (cervical carcinoma) cells and tsa201 cells (European Collection of Cell Cultures); Anti-SNAP-25, anti-FLAG resin, FLAG peptide, Minimum essential medium (MEM), okadaic acid (OA); Trypsin/EDTA, l-glutamine, foetal bovine serum, FM 2–10 styryl dye and SIGMA FAST Protease Inhibitor Cocktail Tablets, EDTA-Free (Sigma-Aldrich, St. Louis, Missouri, USA); [^32^P]ATP[γP] (Hungarian Isotope Institute, Budapest, Hungary), CM5 sensor chip, N-ethyl-N’-dimethylaminopropyl-carbodiimide, N-hydroxysuccinimide, Amine Coupling Kit (Biacore AB, Uppsala, Sweden). All other chemicals used were purchased commercially in the highest purity available.

### Proteins

Proteins were purified as described in previous publications: Skeletal muscle PP1c and PP2Ac^12^, FLAG-tagged alpha isoform of PP1c (rPP1cα)^21^, Hexahistidine-tagged delta isoform of PP1c (rPP1cδ)^22^), Hexahistidine-tagged protein phosphatase 1 inhibitor 2 (I2)^21^, ^32^P-labelled 20 kDa light chain of turkey gizzard myosin (^32^P-MLC20) phosphorylated to an extent of 0.85–0.95 mol phosphate/mol MLC20^23^. Cells of tsA201 were transfected with FLAG-peptide (Asp-Tyr-Lys-Asp-Asp-Asp-Asp-Lys)-coupled myosin phosphatase target subunit 1 (MYPT1, *PPP1R12A*) plasmid and FLAG-MYPT1 as well as its associated proteins (such as PP1cδ) were isolated from cell lysates on anti-FLAG coupled resin. The resin was washed three times with Tris-buffered saline (TBS) and the FLAG-MYPT1-PP1cδ complex was eluted from the resin with 300 µg/ml FLAG-peptide in TBS.

### Assay of protein phosphatase

Protein phosphatase activity of either skeletal muscle PP1c or PP2Ac (1.9–2.2 nM), or rPP1cα or rPP1cδ (2.71–3.72 nM), or FLAG-MYPT1-PP1c (∼9 nM) was assayed at 30 °C in absence or presence of polyphenol compounds (0.1–100 µM) in 20 mM Tris/HCl (pH 7.4), 0.1% 2-mercaptoethanol (30 µL). The purified phosphatase samples or HeLa lysates in 300-fold dilution (1.8–3.6 µg/ml) in the presence or absence of 2 µM I2 were preincubated with increasing concentration of the polyphenol compounds (0–100 µM) for 10 min then the reaction was started by the addition of 1 µM ^32^P-MLC20 and incubated for 10 min. Polyphenol-treated HeLa cells were washed then lysed in 50 mM Tris/HCl (pH 8.0), 150 mM NaCl, 1% (V/V) Triton X-100, 0.15% (w/v) protease inhibitor cocktail. The phosphatase activity was assayed with 1 µM ^32^P-MLC20 for 30 s in 3-fold final dilution (0.24–0.43 mg/ml). The reactions were terminated by the addition of 200 µL of 10% trichloroacetic acid and 200 µL of 6 mg/mL BSA solution. The samples were centrifuged for 3 min at 10,000 rpm and the supernatants (370 µL) were transferred into scintillation vials and the radioactivity of the released ^32^Pi was determined in Tri-Carb 2800TR scintillation counter.

### Cell culturing and treatments

HeLa cells and tsa201 cells were cultured as recommended by the supplier. Prior to treatments HeLa cells were serum starved in MEM medium supplemented with 2 mM L-glutamine for 17 h. For phosphatase activity assay cells were treated with increasing concentration of polyphenol compounds (1–50 µM) for 1 h. To assess cell viability by Alamar-blue assay serum starved cells were treated with ellagitannins (1–50 µM) for 24 h in serum free MEM medium. The media was exchanged for resazurin (20 µM) containing serum free media and the cells were incubated for 2 h to enhance resorufin formation by the viable cells. The emission of resorufin was measured by a fluorimeter at 620 nm.

### Synaptosome preparation and treatments

Preparation of synaptosomes from murine brain cortex was carried out as described previously^24^. Functional synaptosomes were kept in Krebs buffer (118 mM NaCl, 5 mM KCl, 25 mM NaHCO_3_, 1 mM MgCl_2_, 10 mM d-glucose, pH 7.4). After preparation, synaptosomes were treated with either tellimagrandin I or mahtabin A in 1, 10 or 100 µM final concentrations, or with 10 µM tautomycetin (TMC) for 45 min at 30 °C. After the treatments synaptosomes were applied for exocytosis assay or were lysed and the protein lysates were subjected to Western blot. For exocytosis assay an already established protocol^25^ was used. FM 2–10 water-soluble styryl dye was incorporated first into the outer leaflet of synaptosome membrane, then into the synaptosomes. Exocytosis was induced by addition of 30 mM KCl to the synaptosomes. The fluorescent intensity of FM 2–10 styryl dye negatively correlated with the extent of synatposomal exocytosis and was measured by Fluoroskan FL (excitation: 488 nm, emission: 540 nm). Western blot of synaptosome proteins was carried out as described previously^24^^,^^25^.

### Surface plasmon resonance (SPR)

The interaction between rPP1cδ and tellimagrandin I was investigated by surface plasmon resonance based binding technique using Biacore 3000 instrument. rPP1cδ catalytic subunit was immobilised on the surface of CM5 sensor chip by amine coupling protocol as provided by the manufacturer. The sensor chip surfaces were activated by the injection of 35 µL of 50 mM N-hydroxysuccinimide and 200 mM N-ethyl-N’- dimethylaminopropyl-carbodiimide solution for 7 min at a flow rate of 5 µl/min. rPP1cδ was then diluted to 5 µM in the immobilisation buffer containing 10 mM sodium acetate (pH 5.5), 1 mM dithiothreitol, 2 mM MnCl_2_, and 5 µM okadaic acid (OA). OA was given to prevent immobilisation via the substrate-binding site^12^. rPP1cδ was injected over the surface for 7 min at a flow rate of 10 µl/min. Excess reactive sites on the surface were then blocked with a 7-min injection of 1 M ethanolamine/HCl (pH 8.5) at a flow rate of 5 µl/min. Ethanolamine blocked surface was used as reference. Tellimagrandin I was diluted to a concentration range of 0.5–2.5 µM in the running buffer (20 mM Tris/HCl (pH 7.4), 150 mM NaCl, 1 mM ditiotreitol, 2 mM MnCl_2_, 0.05% Surfactant P20) and injected over the control and rPP1cδ surfaces. The association phase of the interaction was monitored for 7 min while the dissociation phase for 6 min. The sensor chip was regenerated after each binding assay by an injection of 10 mM glycine–HCl (pH 2.1). The binding of the analyte to rPP1cδ was recorded as sensorgrams expressed in response units (RU) and plotted as a function of time (in seconds). The non-specific binding of tellimagrandin I was determined by a control surface which was treated identically with the rPP1cδ surface and used to gain normalised binding for tellimagrandin I-rPP1cδ interaction by subtraction of values for non-specific binding. The kinetic and the association/dissociation parameters were derived from the sensorgrams with BiaEvaluation 3.1 software (Biacore AB, Uppsala, Sweden).

### Microscale thermophoresis (MST)

Microscale thermophoresis (MST) experiments were performed on either, a Monolith NT.115 (MST Power: 20%, LED Power: 90%) or on a Monolith NT.LabelFree (MST power: 60%; LED Power: 80%) instruments (NanoTemper Technologies GmbH, Munich, Germany). Measurements on the Monolith NT.LabelFree were performed with 250 nM of unlabelled, recombinant rPP1cα per sample. Measurements on the Monolith NT.115 were performed with 170 nM rPP1cα labelled with NT-647 (NHS labelling, NanoTemper Technologies GmbH, Munich, Germany) per sample. All measurements were performed in MST buffer containing of 25 mM Tris/HCl pH 7.4, 150 mM NaCl, 1 mM TCEP, 0.1%(m/V) PEG 8000.

A 16-point, two-fold dilution series (in MST buffer) of tellimagrandin I, EGCG,or PGG was mixed with non-labelled (for tellimagrandin I and PGG) or labelled (for EGCG) rPP1cα to generate a concentration series of ligands ranging from 120 µM to 7.32 nM. The solutions were loaded into Zero Background Premium Coated or Premium Coated glass capillaries, for the measurement with NT.LabelFree or NT.115 instrument, respectively. Capillaries were placed on the capillary tray and after the capillary scan the change in either intrinsic (NT.LabelFree) or extrinsic (NT.115) fluorescence of rPP1cα upon ligand binding was measured. Data were analysed using MO.AffinityAnalysis software (NanoTemper Technologies GmbH, Munich, Germany).

### Isothermal titration calorimetry (ITC)

Isothermal titration calorimetry (ITC) experiments were carried out at 25 °C by a MicroCal ITC200 instrument. The assay buffer contains 25 mM Tris/HCl (pH 8.0), 100 mM NaCl, and 1 mM DTT. rPP1cα was dialysed in the assay buffer two times for two hours freshly prior to measurements and diluted to 5–10 µM then transferred into the sample cell of the calorimeter. Tellimagrandin I was diluted to 60–100 µM in the assay buffer and measured into the syringe. 18 times 2 µL ligand was then injected into the cell at a stirring rate of 300 rpm. The reference power was 5 µcal/sec and the assay buffer was loaded in the reference cell. The titration data were analysed with MicroCal ITC-Origin software using one sets of site model to obtain binding parameters of ligand-protein interaction.

### NMR saturation transfer difference (STD)

NMR experiments were performed as previously described^12^ with a Bruker Avance II 500 spectrometer operating at 500 MHz and equipped with a 5-mm z-gradient multinuclear proton detection (bbi) probe head. Both ^1^H-NMR and STD spectra were recorded at 293 K on samples containing 0.48 mg of tellimagrandin I dissolved in 500 µL of D_2_O phosphate buffer (pH 6.5) resulting in about 1.2 mM final concentration of tellimagrandin I. The samples for STD binding studies contained about 10 µM of rPP1cδ or rPP1cα. Similar spectra were obtained with both rPP1c isoforms.

### Statistical analysis

Normalized data of multiple groups were analysed with analysis of variance (ANOVA). All data sets were distributed normally. We applied the Holm-Sidak procedure for multiple comparison. Tests were carried out with SigmaPlot software for Windows. The presented data sets are means ± SD.

## Results

### Inhibition of PP1 and PP2A by ellagitannins

**Figure 2.**
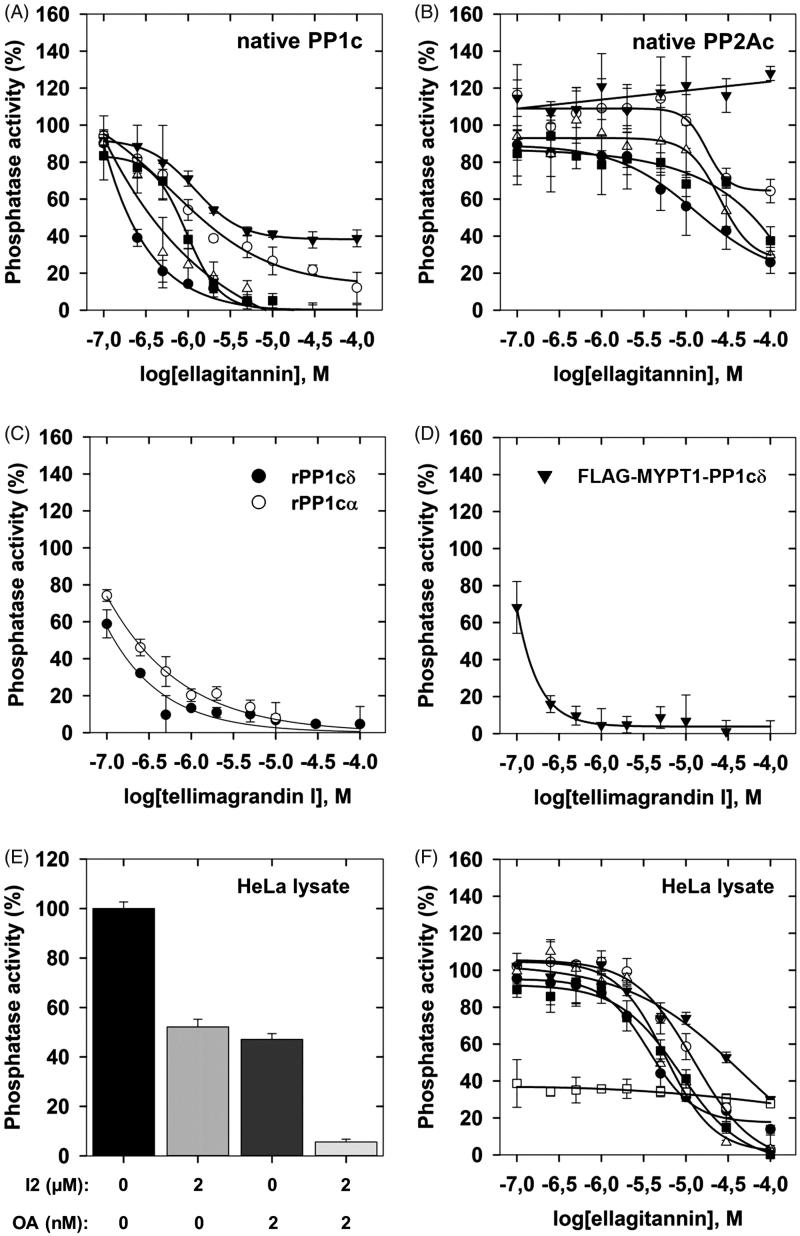
The effect of ellagitannins on the activity of PP1 and PP2A. Ellagitannins (0.1–100 µM) were preincubated with the phosphatase samples for 10 min and the phosphatase activity was determined in triplicates with 1 µM ^32^P-MLC20 substrate as described in “Materials and methods” section. Phosphatase activity was determined in the presence of tellimagrandin I (●); mahtabin A (Δ); praecoxin B (■); GHG (^); pedunculagin (▼) with PP1c (A), PP2Ac (B) or HeLa lysate (F). The effect of tellimagrandin I on the activity of rPP1cα (^) and rPP1cδ (●) (C), or FLAG-MYPT1-PP1cδ (▼) (D), or on the phosphatase activity of HeLa lysate in the presence of I2 (□) (F). The effect of I2 and OA on the phosphatase activity of HeLa lysate (E). Phosphatase activity in the absence of ellagitannins, I2 or OA was taken as 100%. Data are means ± SD (*n* = 3–5).

The ellagitannins (Figure 1) assayed on the activity of PP1c or PP2Ac in this study are similar to PGG in composition, but they differ structurally in two important aspects: (i) all of them include hexahydroxydiphenoyl unit linked in various positions, except for pedunculagin which includes two of these linkages; (ii) tellimagrandin I, pedunculagin, and praecoxin B have free glycosidic hydroxyls, while GHG has an unmodified hydroxyl at position 3 of the glucopyranose ring. These molecules exerted distinct inhibitory influence on native PP1c and PP2Ac. Figure 2(A,B) illustrate the concentration dependent inhibitory effectiveness of ellagitannins on native PP1c and PP2Ac purified from rabbit skeletal muscle, respectively. Table 1 shows the IC_50_ values determined for PP1c and PP2Ac, which indicate that PP1c is more sensitive to inhibition by these ellagitannins than PP2Ac. This is also reflected in the PP1/PP2A selectivity ratio (Table 1) of 1:98, 1:82, and 1:81 for tellimagrandin I, mahtabin A, and praecoxin B, respectively, the three most potent ellagitannin inhibitors. We established that the purified, recombinant isoforms of PP1cα and PP1cδ (rPP1cα and rPP1cδ) were inhibited by tellimagrandin I (Figure 2(C)) with IC_50_ values of 0.23 ± 0.05 µM and 0.13 ± 0.02 µM, respectively, which are similar to that of the native PP1c. Figure 2(D) shows that tellimagrandin I inhibits myosin phosphatase holoenzyme consisting of the FLAG-MYPT1-PP1cδ complex with similar potency (IC_50_=0.11 ± 0.02 M) to that of the isolated PP1c catalytic subunits. Inhibition of the phosphatase activity by ellagitannins in HeLa cell lysate was also assessed. First, we determined the activity of PP1 and PP2A in the lysates using I2 to inhibit PP1, and low concentration of OA (2 nM) to suppress PP2A specifically, in order to determine the contribution of PP1 and PP2A to the phosphatase activity of lysates (Figure 2(E)). These data suggest that PP1 (∼48%) and PP2A (∼51%) activities distribute in the HeLa lysate at approximately equal ratio. We found that although the inhibitory effectiveness of ellagitannins on the phosphatase activity of HeLa lysate followed the same order as with the purified enzymes, the IC_50_ values were at least one order of magnitude higher (Table 1). These higher IC_50_ values might be due to that holoenzymes of both PP1 and PP2A were assayed in the lysate simultaneously with their different sensitivities. As the myosin phosphatase holoenzyme appears to be similarly sensitive to inhibition by tellimagrandin I as PP1c (Figure 2(D)) the lower sensitivity to inhibition in the lysates may not be due to interaction of the catalytic subunits with regulatory proteins. However, the lower sensitivity of PP2A may be involved as after suppression of PP1 activity by I2 in the lysate the phosphatase activity due to PP2A was inhibited by tellimagrandin I slightly even at the highest concentration applied (Figure 2(F)). On the other hand, the quite “sticky” nature of polyphenols allows binding to several proteins in the lysate decreasing the effective concentration of the polyphenols for inhibition as previously discussed^12^.

**Table 1.** IC_50_ values for the inhibition of PP1 and PP2A by ellagitannins.

	IC_50_ (µM)			PP1c/PP2Ac
Ellagitannins	PP1c	PP2Ac	HeLa lysate	selectivity
tellimagrandin 1	0.20 ± 0.02	19.52 ± 8.60	4.46 ± 0.95	1:97
mahtabin A	0.41 ± 0.18	33.77 ± 8.44	6.19 ± 0.37	1:82
praecoxin B	0.79 ± 0.11	63.86 ± 5.52	6.97 ± 1.12	1:80
GHG	1.41 ± 0.30	>100	12.97 ± 3.30	n.d.
pedunculagin	2.47 ± 0.22*	>100	33.90 ± 4.54	n.d.

The IC_50_ values were derived from the data of Figure 2(A,B,E) and given as means ± SD.

n.d.: not determined.

* Approximate value determined from partial inhibition.

### Interaction of polyphenols with PP1c

**Figure 3.**
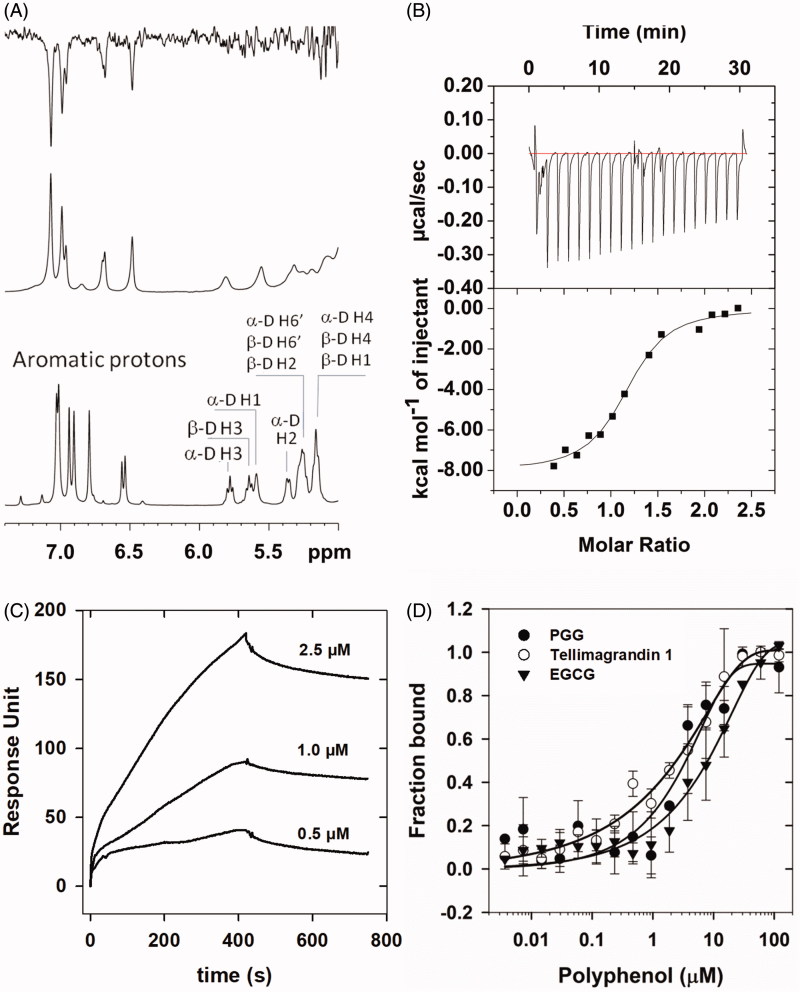
Interaction of PP1c with tellimagrandin I and with other polyphenols. (A): NMR spectra of tellimagrandin I in the absence (lower panel) and in the presence of rPP1cα (middle panel); and the corresponding STD-NMR spectrum (upper panel). (B): Interaction of rPP1cα with tellimagrandin I as revealed by ITC: ΔH = 8.038 ± 0.275 kcal/mol; S = 0.0036 kcal/mol/K; *N* = 1.16 ± 0.03; K_a_=4.68 ± 1.02x10^6^ M ^− 1^. (C): Interaction of rPP1cδ with tellimagrandin I as revealed by SPR: K_d_=0.31 µM. Representative sensorgrams of two independent experiments are shown. (D): Affinities of rPP1cα to tellimagrandin I (^), PGG (●) and EGCG (▼) were measured using MST. The changes in either, the intrinsic fluorescence of unlabelled rPP1cα upon binding to tellimagrandin I and PGG, or changes in fluorescent signal from rPP1cα extrinsically labelled with NT647 dye upon binding with EGCG, were determined at a range of concentration of polyphenols, and the fractions bound are presented. Data points are means ± SD (*n* = 3).

Experiments with tellimagrandin I and rPP1cα or rPP1δ were carried out to assess their interaction with the application of different binding techniques (Figure 3). ^1^H NMR spectra of tellimagrandin I recorded in the absence (Figure 3(A), bottom spectrum) and in the presence of 10 µM rPP1cα (Figure 3(A), middle spectrum) show remarkable differences. The significant broadening of ^1^H resonance signals of tellimagrandin I observed in the presence of rPP1cα (Figure 3(A), middle spectrum) is a strong indication of the chemical exchange between the free and PP1c-bound form of tellimagrandin I. One-dimensional (1D) ^1^H NMR saturation transfer difference (STD) experiment provided further evidence and structural details on the formation of tellimagrandin I-rPP1c complexes. The STD NMR spectrum (Figure 3(A), top spectrum) identifies the aromatic ring hydrogens in close contacts with PP1c suggesting that hydrophobic interactions may dominate the binding of tellimagrandin I to PP1c. Based on the similarity of the interaction of PP1c and tellimagrandin I to that of PGG^12^ as well as on the potent inhibitory influence of tellimagrandin I on PP1c, it may be concluded that tellimagrandin I might also occupy part of the hydrophobic substrate binding groove on the surface of PP1c.

PP1c-tellimagrandin I interaction was also assessed by isothermal titration calorimetry (ITC) and surface plasmon resonance based (SPR) binding techniques (Figure 3(B,C)) and the dissociation constants (K_d_) determined were 0.23 µM and 0.31 µM, respectively. These K_d_ values were in good agreement with the IC_50_ value (0.20 µM) determined for inhibition of native PP1c by tellimagrandin I (Table 1). We also applied microscale thermophoresis (MST) based technique to compare binding affinity of different polyphenols (tellimagrandin I, PGG, and EGCG) to PP1c. MST determined K_d_ values for the interaction of PP1c with tellimagrandin I, PGG, and EGCG were 1.78 ± 0.59 µM, 3.08 ± 0.35 µM and 8.2 ± 4.3 µM, respectively. The affinities of the polyphenols to PP1c follow the same order as their inhibitory potencies reflected in the IC_50_ values (Table 1)^12^.

### Effect of polyphenols on cellular systems

**Figure 4.**
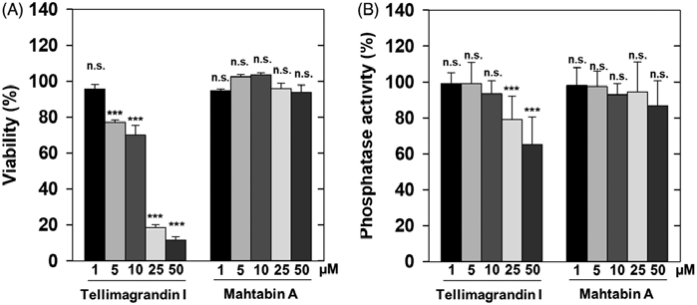
Distinct influence of ellagitannins on the survival and phosphatase activity of HeLa cells. The effect of tellimagrandin I and mahtabin A was assayed on the survival (A) and the phosphatase activity (B) of HeLa cells in 0–50 µM concentration range as detailed in Materials and methods. The cell survival and the phosphatase activity in the absence of ellagitannins were taken as 100%. Data represent means ± SD (*n* = 3). ANOVA *p < .05, **p < .01, ***p < .001, n.s.: not significant.

Previous results provided data for the effects of tellimagrandin I on cells and revealed that differentiation of human leukemic K562 cells^26^ and gap junctional communication of HeLa cells were influenced by this polyphenol^27^. However, no attempts were made to relate these effects to changes in phosphatase activities. We tested first the effect of tellimagrandin I and mahtabin A on the survival and on the phosphatase activity of HeLa cells. Tellimagrandin I suppressed the viability of HeLa cells dramatically in 5–50 µM concentration range after 24 h incubation (Figure 4(A)). Tellimagrandin I partially reduced the phosphatase activity of HeLa cells (after 1 h incubation) in a concentration dependent manner and the effective concentration range was 10–50 µM (Figure 4(B)). These data may suggest that phosphatase inhibition by tellimagrandin I contributes to the initiation of cell death of HeLa cells, however, the different time courses of the viability and phosphatase assays as well as the lack of revealing more detailed molecular background make this conclusion elusive. Quite surprisingly mahtabin A, which is similar in structure to tellimagrandin I and it is also among the potent ellagitannin inhibitors of PP1 (Table 1), was without effect on either the survival or the phosphatase activity of HeLa cells (Figure 4(A,B)).

**Figure 5.**
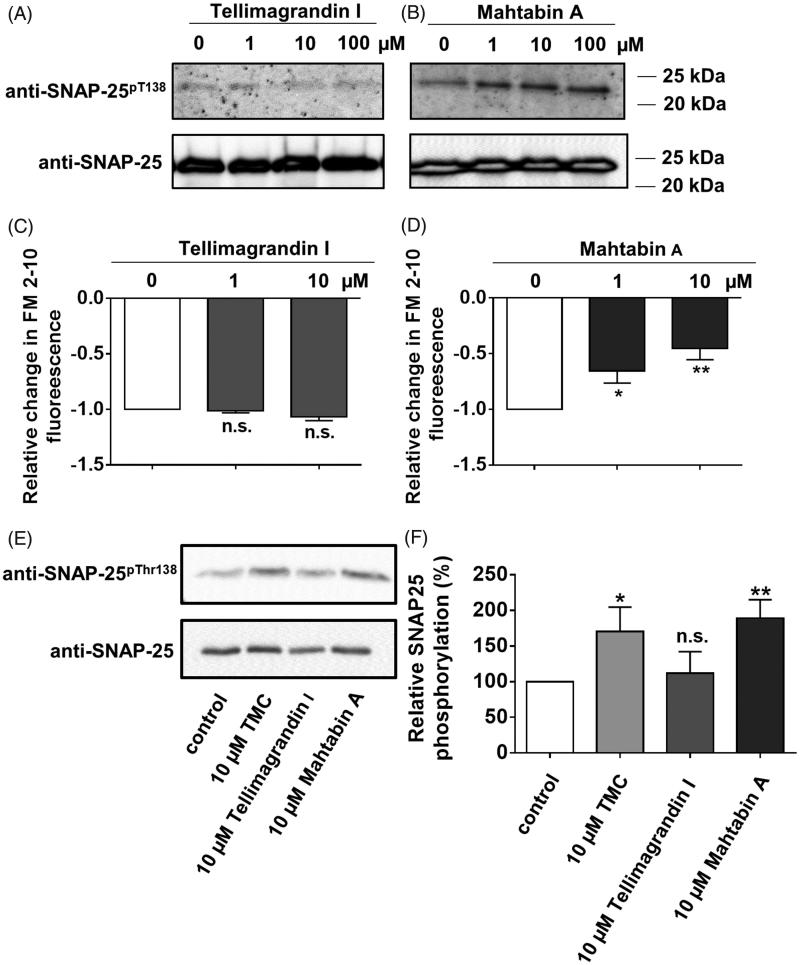
Effect of tellimagrandin I and mahtabin A on the phosphorylation of SNAP-25^Thr138^ and on the exocytosis of mouse synaptosomes. The influence of tellimagrandin I (A and C) and mahtabin A (B and D) on the phosphorylation of SNAP-25^Thr138^ (A, B), and on the exocytosis of synaptosomes (C, D). Comparison of the effect of tellimagrandin I, mahtabin A and TMC on the phosphorylation level of SNAP-25^Thr138^. E: Representative Western blot with anti- SNAP-25^pThr138^ specific antibody. F: Relative changes in the amount of SNAP-25^pThr138^ determined by densitometry of the Western blots normalised on the bands obtained for the loading controls. Data are means ± SD (*n* = 3). ANOVA *p < .05, **p < .01, ***p < .001, n.s.: not significant.

To further elucidate the possible physiological influence of tellimagrandin I and mahtabin A we studied their effect on the exocytosis of mouse cortical synaptosomes that is an *ex vivo* preparation with intact membranes and serves as a model for studies of exocytosis/neurotransmitter release^25^. In our previous report we described that synaptosome exocytosis is inversely correlated with the increased phosphorylation of synaptosomal-associated protein of 25 kDa (SNAP-25) at Thr138 (SNAP-25^Thr138^) in a RhoA-associated protein kinase/myosin phosphatase (PP1-type) mediated manner^24^. First, we tested how incubation of synaptosomes with tellimagrandin I (Figure 5(A)) or mahtabin A (Figure 5(B)) influenced the phosphorylation level of SNAP-25^Thr138^. Tellimagrandin had no any effect while mahtabin A increased the amount of phosphorylated SNAP-25^Thr138^ (SNAP-25^pThr138^) in a concentration dependent manner at a range of 1–100 µM. Second, the effect of these polyphenols on KCl induced synaptosomal exocytosis was probed and the results revealed that mahtabin A suppressed exocytosis measured as a decrease in FM 2–20 fluorescence (Figure 5(D)), whereas tellimagrandin I was without any influence (Figure 5(C)). Finally the effect of tellimagrandin I and mahtabin A was compared to tautomycetin (TMC), a well-defined PP1 specific inhibitor in synaptosomes^25^. Figure 5(E,F) demonstrate that TMC and mahtabin A increased the amount of SNAP-25^pThr138^ in the same manner while tellimagrandin I had no influence. These data support the conclusions that the two ellagitannins, in spite of their similar structures and phosphatase inhibitory features, mediate the physiological responses of cells as well as membrane-surrounded *ex vivo* preparations in diverse manners.

## Discussion

Our present study establishes ellagitannins as inhibitors of both PP1 and PP2A with their higher preference for inhibition of PP1. These results are in accord with our previous data suggesting that the gallotannic PGG differentiated between these two phosphatase types^12^ similarly, however, its selectivity was lower compared to ellagitannins (Table 1). These differences could be due to the distinct structural features of the ellagitannins and PGG. The structures of the five ellagitannins assayed in this study differ from PGG in having one (tellimagrandin I, mahtabin A, praecoxin B, GHG) or two (pedunculagin) covalently linked hexahydroxydiphenoyl units in various positions, and except for mahtabin A they have also one unesterified hydroxyl on the glucopyranose ring (Figure 1). The structure-inhibitory potency relationship of ellagitannins on the activity of PP1c suggest that the position of the linkages of the hexahydroxydiphenoyl units may be an important determinant in the inhibitory effectiveness of the ellagitannins. Compared to PGG tellimagrandin I represents a more rigid structure due to the 4,6 hexahydroxydiphenoyl unit and it is a more potent inhibitor of PP1c. Tellimagrandin I also includes an unesterified glycosidic hydroxyl, and a lack of the galloyl unit in position 1 may be another factor which contribute to the increased inhibitory potency. With the latter regard mahtabin A, in which all hydroxyls are esterified, exerts weaker inhibitory potency (similar to PGG) than tellimagrandin I, although it is difficult to disclose if this difference is due to the esterification of glycosidic hydroxyl or the presence of a distinct, 2,3 hexahydroxydiphenoyl unit in mahtabin A. Our data favour the importance of the positions of the hexahydroxydiphenoyl linkages as praecoxin B being different from tellimagrandin I only in this linkage (2,3 positions) has lower inhibitory potency than tellimagrandin I. On the other hand, GHG has 4,6 hexahydroxydiphenoyl unit like in tellimagrandin I, but it is esterified with gallic acid at the glycosidic hydroxyl and lacks galloyl units at position 3. Because of the latter two structural differences GHG is a less powerful inhibitor than tellimagrandin I or praecoxin B. These results emphasise the importance of the presence of a galloyl unit at position 3 for effective inhibition and argues against the significance of the esterification of the glycosidic hydroxyl in the inhibition. Moreover, having two hexahydroxydiphenoyl linkages in the molecule, like in pedunculagin, significantly reduces the inhibitory potency.

The above data all together suggest that moderately increased rigidity in the ellagitannins by formation preferably a 4,6 hexahydroxydiphenoyl unit is in favour of higher phosphatase inhibitory potency, but certain flexibility of the ellagitannin molecules are still required to exert effective inhibitory influence. The latter is justified by the effect of pedunculagin which represent a more rigid structure than the other ellagitannins with two hexahydroxydiphenoyl units and proved to be the weakest inhibitor. Based on NMR STD analysis the aromatic rings of the galloyl units may play an important role in the interaction of ellagitannins with the hydrophobic groove of the substrate binding pocket of PP1c. Previous results suggested that the hydroxyls of the galloyl or flavanol units, as in case of PGG or EGCG, participated also in strengthening the interaction toward PP1c by forming multiple hydrogen bonding^12^. This was not verified experimentally here with the ellagitannins, but phenolic hydroxyls in polyphenolic compound are suggested to bind to many protein targets^28^, therefore it is reasonable to assume that the galloyl hydroxyls in ellagitannins can also be involved in similar interaction patterns with PP1c. It is to note, however, that while interactions of the ellagitannins and their inhibitory features are quite similar to PGG (and possibly to other gallotannins) they exert much lower inhibitory influence on PP2Ac than PGG. It is reflected in the higher selectivity ratio of ellagitannins (1: 80/97) for PP1c: PP2A compared to that of PGG (1:17). Reasons for these findings are not clear yet, however, this difference might be due to the limited availability of the hydrophobic surface of PP2A for the ellagitannin molecules, similar to that was hypothesised for the weak interaction of PP2Ac with EGCG based on the 3 D structure of PP2Ac^29^ and molecular docking studies^12^.

We have proven the interaction of tellimagrandin I and PP1c by four independent methods, NMR STD, SPR, ITC, and MST measurements. While NMR STD data reveal the structural features of the interaction, the ITC, SPR, and MST methods are able to quantify the strength of the binding and provide association/dissociation constants for the PP1c-tellimagrandin I complex. The K_d_ values for this interaction ranged from 0.23 µM to 1.78 µM depending upon the applied measurement methods. It should be noted that the phenomenon of stacking of ellagitannins on the surface of PP1c is also considered here as it was suggested for PGG and EGCG^12^^,^^30^ in SPR measurement. This is indicated by the higher response unit values (Figure 3(C)) than expected from a simple 1:1 interaction model used here to derive the K_d_ value. Although stacking cannot be excluded in case of ITC or MST either but it might be different in these assays since both of the binding partners are in solution and neither of them is surface-immobilised. These differences might explain the range of K_d_ values obtained by the different measurement methods. Nevertheless, the affinity of binding of the different polyphenols (tellimagrandin I, PGG, EGCG) to PP1c reflects the order of PP1 inhibitory potency.

Ellagitannins have been reported to influence numerous biological processes and their effects have also been proven therapeutic values as reviewed in Ismail et al.^31^ on several types of cancer cells. We have found that tellimagrandin I reduced both the viability and the phosphatase activity of HeLa cells, while mahtabin A quite surprisingly was without effect. As mahtabin A was an effective phosphatase inhibitor in HeLa cell lysate the lack of influence either on viability or on cellular phosphatase activity is most probably due to its inability to permeate through the cell membrane. However, the effect of these ellagitannins in cellular systems still remains to be controversial. Thus, mahtabin A increases the phosphorylation of SNAP-25, an internal substrate in mouse cortical synaptosome, similarly as does TMC, a PP1 specific inhibitor, but here tellimagrandin I is without influence. Moreover, mahtabin A and tellimagrandin I act differently on the physiological responses of synaptosomes as mahtabin A suppresses KCl induced exocytosis while tellimagrandin I has no influence. The *ex vivo* preparation of synaptosomes is considered to be membrane surrounded and mahtabin A is believed to penetrate into the synaptosomes to inhibit internal PP1 and to increase the phosphorylation level of SNAP-25, an internal substrate. In contrast, tellimagrandin I does not seem to carry the above features and therefore is without effect in synaptosomes. It is not understood yet why two structurally similar compounds act so differently depending upon the cellular system. However, it appears to be certain that ellagitannins can inhibit intracellular P-Ser/Thr specific phosphatases thereby they could regulate phosphorylation events and coupled cellular processes.

In conclusion, ellagitannins represent a novel family of compounds that are able to inhibit PP1 and PP2A distinctly with preferential suppression of PP1. Quantitative binding analyses indicate a medium stability for the complex of PP1 with tellimagrandin I and there are indications that the interaction occurs via the hydrophobic substrate binding groove of PP1c and the aromatic rings of tellimagrandin I. Intracellular targets of ellagitannins have not been widely identified so far. Our present data identify PP1 as a preferred target of ellagitannins in cells suggesting that these polyphenols may control the phosphorylation level of many proteins, which are dephosphorylated by PP1. These data imply that regulation of the phosphorylation-dephosphorylation processes is also involved in the numerous cellular influences of ellagitannins.
